# Galvanic vestibular stimulation in hemi-spatial neglect

**DOI:** 10.3389/fnint.2014.00004

**Published:** 2014-01-29

**Authors:** David Wilkinson, Olga Zubko, Mohamed Sakel, Simon Coulton, Tracy Higgins, Patrick Pullicino

**Affiliations:** ^1^School of Psychology, University of KentCanterbury, UK; ^2^East Kent Neuro-Rehabilitation Service, East Kent Hospitals University NHS Foundation TrustCanterbury, UK; ^3^Centre for Health Services Studies, University of KentCanterbury, UK

**Keywords:** stroke, neuro-stimulation, clinical trial, hemi-inattention, rehabilitation

## Abstract

Hemi-spatial neglect is an attentional disorder in which the sufferer fails to acknowledge or respond to stimuli appearing in contralesional space. In recent years, it has become clear that a measurable reduction in contralesional neglect can occur during galvanic vestibular stimulation, a technique by which transmastoid, small amplitude current induces lateral, attentional shifts via asymmetric modulation of the left and right vestibular nerves. However, it remains unclear whether this reduction persists after stimulation is stopped. To estimate longevity of effect, we therefore conducted a double-blind, randomized, dose-response trial involving a group of stroke patients suffering from left-sided neglect (*n* = 52, mean age = 66 years). To determine whether repeated sessions of galvanic vestibular stimulation more effectively induce lasting relief than a single session, participants received 1, 5, or 10 sessions, each lasting 25 min, of sub-sensory, left-anodal right-cathodal noisy direct current (mean amplitude = 1 mA). Ninety five percent confidence intervals indicated that all three treatment arms showed a statistically significant improvement between the pre-stimulation baseline and the final day of stimulation on the primary outcome measure, the conventional tests of the Behavioral Inattention Test. More remarkably, this change (mean change = 28%, *SD* = 18) was still evident 1 month later. Secondary analyses indicated an allied increase of 20% in median Barthel Index (BI) score, a measure of functional capacity, in the absence of any adverse events or instances of participant non-compliance. Together these data suggest that galvanic vestibular stimulation, a simple, cheap technique suitable for home-based administration, may produce lasting reductions in neglect that are clinically important. Further protocol optimization is now needed ahead of a larger effectiveness study.

## Introduction

Hemi-spatial neglect is a debilitating, attentional disorder that most commonly arises from damage to the right-side of the brain (Robertson and Halligan, 1999). Sufferers fail to acknowledge or respond to visual information presented on the side of space opposite their brain lesion (e.g., the left), and as such struggle with many daily routines, characteristically bumping into obstacles, failing to notice people on the affected side or cleaning only one side of their body. Prevalence is hard to estimate because diagnostic criteria differ, but conservative estimates indicate that of the ~150,000 UK residents who suffer a stroke per year, approximately 18% (i.e., 27,000) will show moderate to severe left-sided neglect in the acute phase, with ~7% (i.e., 10,000) continuing to show stable impairment beyond 3 months (Ringman et al., 2004). Unfortunately, the presence of left neglect is very strongly associated with poor general functional outcome. Individuals with neglect (regardless of severity) typically require additional weeks in hospital (Katz et al., 1999; Wilkinson et al., 2012), need nearly twice as many hours of physiotherapy and occupational therapy, and are more prone to falls and persistent urinary incontinence (Paolucci et al., 2001). Compared to others with the same Barthel Index (BI) score at hospital admission, patients with neglect score significantly lower on measures of functional independence both during hospital stay and 18 months after leaving (Jehkonen et al., 2000; Gillen et al., 2005; Nijboer et al., 2013). Those who still show neglect on simple bedside tests 2 months after admission have a higher risk of functional worsening at 1 year follow-up. Post discharge, patients with neglect are more likely to require ambulatory assistance and long-term institutionalization or assisted living Kalra et al., 1997; Katz et al., 1999; Nijboer et al., 2013.

Regrettably, many cases of neglect are refractory to treatment. According to a Cochrane Review conducted in 2013, “the effectiveness of rehabilitation strategies for reducing the disabling effects of neglect and increasing independence remains unproven,” (Bowen et al., 2013, p. 1). The review pointed out that although several new treatment approaches meet proof-of-concept, too few studies have progressed these to the level of randomized, controlled trials.

Near complete, but transient, relief from neglect has been observed during artificial stimulation of the vestibular nerves (Rubens, 1985; Cappa et al., 1987). These nerves send information from the semi-circular canals and otoliths of the inner ear to, among other brain regions, parts of the peri-sylvia involved in spatial attention and awareness (Suzuki et al., 2001; Balaban et al., 2011). The conventional method, caloric vestibular stimulation, involves the injection of thermal current (usually via cold water) into the ear canal. This alters the density of endolymph within the nearby balance organs and in turn modulates their afferent firing patterns (see Miller and Ngo, 2007). Unfortunately, the therapeutic benefit of CVS is offset by severe vertigo, nausea and the more general impracticality of ear irrigation, all of which hinder repeated use.

Recent studies suggest that a related technique, known as galvanic vestibular stimulation may provide a more tolerable and simpler way of harnessing this beneficial effect (see Utz et al., 2010). GVS involves the delivery of tiny electrical currents via two small electrodes to the mastoid processes which overlie the vestibular nerves (Coats, 1972). The currents modulate the firing rates of the vestibular nerves in a similar manner to natural head movement, inducing broad-scale compensatory responses across cortical and subcortical regions (Bense et al., 2001; Wilkinson et al., 2012). The electric currents are applied at a level (~1 mA) that is too low to be felt by the patient and without the need for patient agency or motivation which are often compromised in neglect.

Preliminary studies show that a single 15–30 min session of GVS improves performance across a range of visuo-spatial tasks including line bisection, figure copying and target cancellation (Rorsman et al., 1999; Wilkinson et al., 2010; Utz et al., 2011a). Several recent studies also hint, but by no means confirm, that the beneficial effects of GVS persist after stimulation is stopped. In an unblinded study performed on two neglect patients, Zubko et al. (2013) showed that a week's programme of GVS was associated with statistically fewer omissions on the star and letter cancellation tasks 3 days post-stimulation. In two other small-group studies conducted on non-neglectors, Kerkhoff and colleagues showed that GVS induced lasting relief for up to 12 weeks from the somatosensory disorder of tactile extinction (Kerkhoff et al., 2011; Schmidt et al., 2013). Given that these studies provide only indirect support for the idea that GVS can induce lasting carry-over from neglect, the need arises for a more reliable estimate of the duration and magnitude of recovery. If, under more tightly controlled and adequately powered conditions, proof of carry-over can be shown then further investigations into the rehabilitative potential of GVS would be warranted.

Most forms of neuro-rehabilitation tend to rely on repeated application to induce carry-over, a finding that chimes with the recent discovery that experience-dependent, long-term plastic change requires multiple stimulus exposures (Hoffman and Cavus, 2002). In the case of hemi-spatial neglect, several techniques other than GVS (e.g., transcranial magnetic stimulation, optokinetic stimulation) have induced gains, albeit of limited scope, for 2 weeks or more following 5–10 consecutive, 30 min daily sessions (Kleinjung et al., 2005; Shindo et al., 2006; Naeser et al., 2012). Similar treatment periods have induced long-term remission from other neuropsychological disorders (McKay et al., 2002; Ohn et al., 2008). These studies suggest that repeated administration not only increases the length of recovery, but also the magnitude of recovery. Contrary to the preliminary data described above (Kerkhoff et al., 2011; Schmidt et al., 2013; Zubko et al., 2013), these studies imply that GVS may be most effective when repeatedly, as opposed to singularly, applied.

The present study had two specific aims: to establish whether (1) GVS can induce a recovery from neglect that lasts beyond the stimulation period, and (2) carry-over is more effectively induced via a single or repeated sessions. To test these hypotheses we allocated, at random, 52 experimental volunteers with left-sided hemi-spatial neglect to one of three treatment arms in which they received 1, 5, or 10 sessions of subliminal GVS, with those in the 1 and 5 treatment arms also receiving 9 and 5 sham sessions respectively. Follow-up tests and questionnaires were conducted 1, 2, and 4 weeks later to assess the severity of neglect symptoms, transfer to activities of daily living, and compliance.

## Materials and methods

### Participants

Participants were recruited between July 2011 and November 2012 from nearby acute stroke and neuro-rehabilitation units in South East England, although a handful of participants self-referred from other parts of the UK following national media coverage. Individuals were eligible if they scored ≤129 on the conventional tests of the Behavioral Inattention Test (BIT) (Halligan et al., 1987); suffered a right unilateral stroke (confirmed by CT or MRI scan); ≥6 weeks post-stroke; ≥18 years; scored ≤2 on the 6-item screener for dementia (Callahan et al., 2002), and scored ≤29 on the Beck Depression Inventory (Beck et al., 1996). Individuals with neglect and suspected visual field loss were included because there is evidence that they can still benefit from GVS (e.g., Rorsman et al., 1999; Wilkinson et al., 2005; Utz et al., 2011a). The presence of hemianopia was not recorded for study purposes because formal field perimetry was not available for many participants. Individuals with titanium plates were also included provided these did not lie beneath or directly adjacent to the stimulation sites. Individuals were excluded if they showed evidence of moderate to severe aphasia on clinical examination and/or prior significant neurological or vestibular illness. Patients with electronic implants, such as cardiac pacemakers, were also excluded given the potential for electrical interference from the vestibular stimulator.

### Recruitment, allocation, and blinding

All participants were informed of the study and provided written informed consent prior to assessment. The study received NHS ethical approval from the London City & East NRES committee, and was conducted in accordance with Medical Research Council (UK) Good Clinical Practice guidelines and the Declaration of Helsinki. Prior to participant enrolment, the trial was registered on the UK Clinical Research Network Study Portfolio Database (UKCRN ID: 10505).

Patients who met eligibility were randomly assigned to one of the three treatment arms (1 active and 9 sham treatments vs. 5 active and 5 sham treatments vs. 10 active and 0 sham treatments) using minimization controlling for age (60 years or more vs. less than 60 years), inpatient/outpatient status, and severity of neglect as measured by the conventional measures of the BIT. Randomization was conducted using a secure, remote randomization facility independent of the research team.

Treatment allocation was double-blind; since the GVS was sub-sensory participants did not know their allocation, and a stimulation protocol (active or sham) pre-determined by the randomization officer was naively administered by the experimenter by typing a 4 digit code (which changed every time) into the stimulation device. Participants' in-patient neglect treatment (typically visual scanning therapy but sometimes limited to the informal reminders given by occupational therapy staff to look left during functional activities) was suspended while they remained on-study. Treatment begun within 1 week of baseline assessment.

### Outcome measures

The primary outcome measure, severity of neglect 4 weeks post-stimulation, was measured using the conventional measures of the BIT. Transfer to activities of daily living was measured using the BI (Mahoney and Barthel, 1965). The BIT and BI were administered by the experimenter at baseline, on the final day of stimulation, and then 1, 2, and 4 weeks post-stimulation. Participant well-being was captured via daily diary cards and an end-of-study satisfaction questionnaire which were completed by the participant often with the help of a relative or friend.

### Treatment preparation

Bipolar, binaural current was delivered through a pair of 2 × 4 cm carbon-rubber, self-adhesive, disposable stimulating electrodes placed over participants' mastoid processes. To ensure complete electrical contact with the electrodes, surrounding skin was cleansed with an alcohol swab and conductive gel coated on the undersides of the electrodes. To induce leftward deviation in the lateral plane, the anode was placed over the left mastoid and the cathode over the right mastoid. The electrodes were connected to a *Magstim Eldith Transcranial DC Stimulator Plus*™ device that was pre-programmed to deliver either 0 or 1 mA mean (0.5–1.5 mA) noisy current for 25 min. Earlier pilot work indicated that older, stroke patients rarely report the presence of a noisy 1 mA DC waveform. In line with this, the incidence with which our participants reported unusual sensations during stimulation, such as pain, tingling or itching behind the ears, were no greater than at the “no stimulation” baseline (see Table 12). Participants were informed that although all participants would receive at least one session of active stimulation, the number of active sessions would vary from participant to participant. During stimulation, participants rested and remained either seated or positioned upright in bed. Stimulation was performed daily from Monday to Friday for two consecutive weeks. All sham sessions were administered first to ensure that, across participants, equal time had elapsed between the final session of active stimulation and the first follow-up assessment. This meant that in the 1 active condition, active stimulation was administered on the final (i.e., 10th) stimulation day, while participants in the 5 active condition received active stimulation from days 6 to 10.

## Results

**Figure 1:**
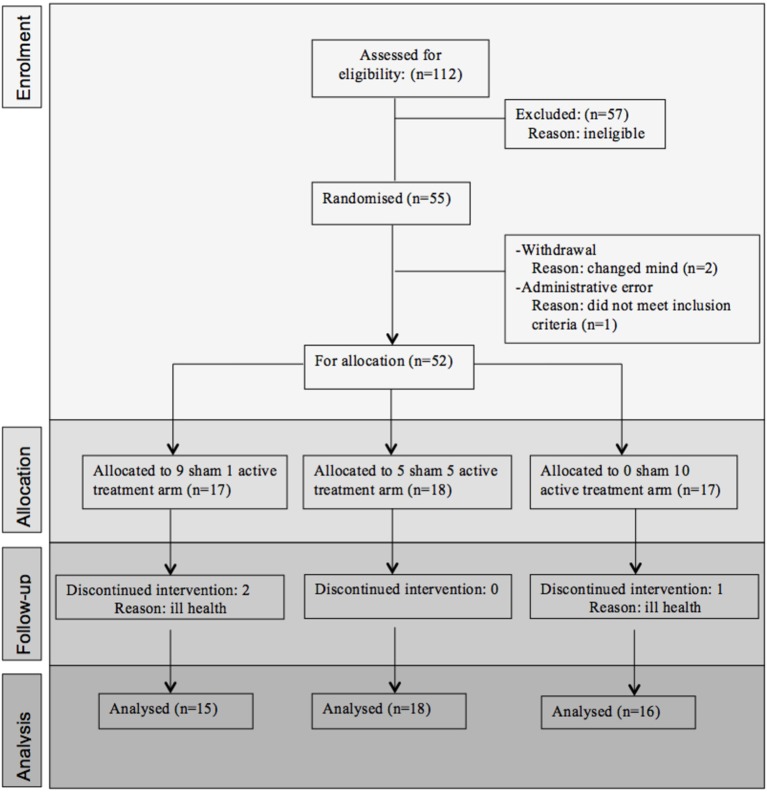
**Consort statement**.

Fifty-five participants were considered eligible, provided consent and randomized (see Figure 1). Of these, 6 participants did not complete the treatment protocol resulting in a total of 49 patients with evaluable data across the three treatment regimens. This number allowed us to meet our target enrolment of 15 participants per treatment arm which was deemed sufficient to allow a potential effect size difference of 0.8 to be detected at 80% power and an alpha of 0.05. The sample demographic characteristics were similar across all three arms (see Table 1).

**Table 1:** **Summary of demographic data**.

		**Treatment regimen**		
		**1 Active**	**5 Active**	**10 Active**
Total *N*		15	18	16
Age (years)	Mean	66.9	66.0	65.7
	*SD*	10.6	9.37	8.72
	Median	66.0	66.5	66.0
	Range	33	38	33
Gender (*N*)	Male	12	12	13
	Female	3	6	3
Ethnicity (*N*)	White European	15	18	16
Patient type (*N*)	Inpatient	10	9	9
	Outpatient	5	9	7
Time since	Median	68	75	94
stroke (days)	Interquartile range	39–229	41–479	39–534

The analysis was conducted as a per protocol analysis, in that only those who completed the intervention and follow-up were evaluated. The primary outcome measure, BIT score at 4 weeks stimulation, was evaluated using an analysis of covariance adjusting for the baseline covariates of BIT score at enrolment, in/out patient status, and age. Analyses of the BI scores were also adjusted for these covariates. Summary data were collated for descriptive analyses of the sub-tests/sub-scales of the BIT and BI, and to show the level of participant satisfaction and the incidence/nature of adverse events. Given the unexpected variation in time since stroke across treatment arm (see Table 1), exploratory analyses were also conducted *post hoc* using the analysis of covariance described above but with Time since Stroke as an additional co-variate to explore its impact on the primary and secondary outcome measures.

### Behavioral inattention test

**Figure 2:**
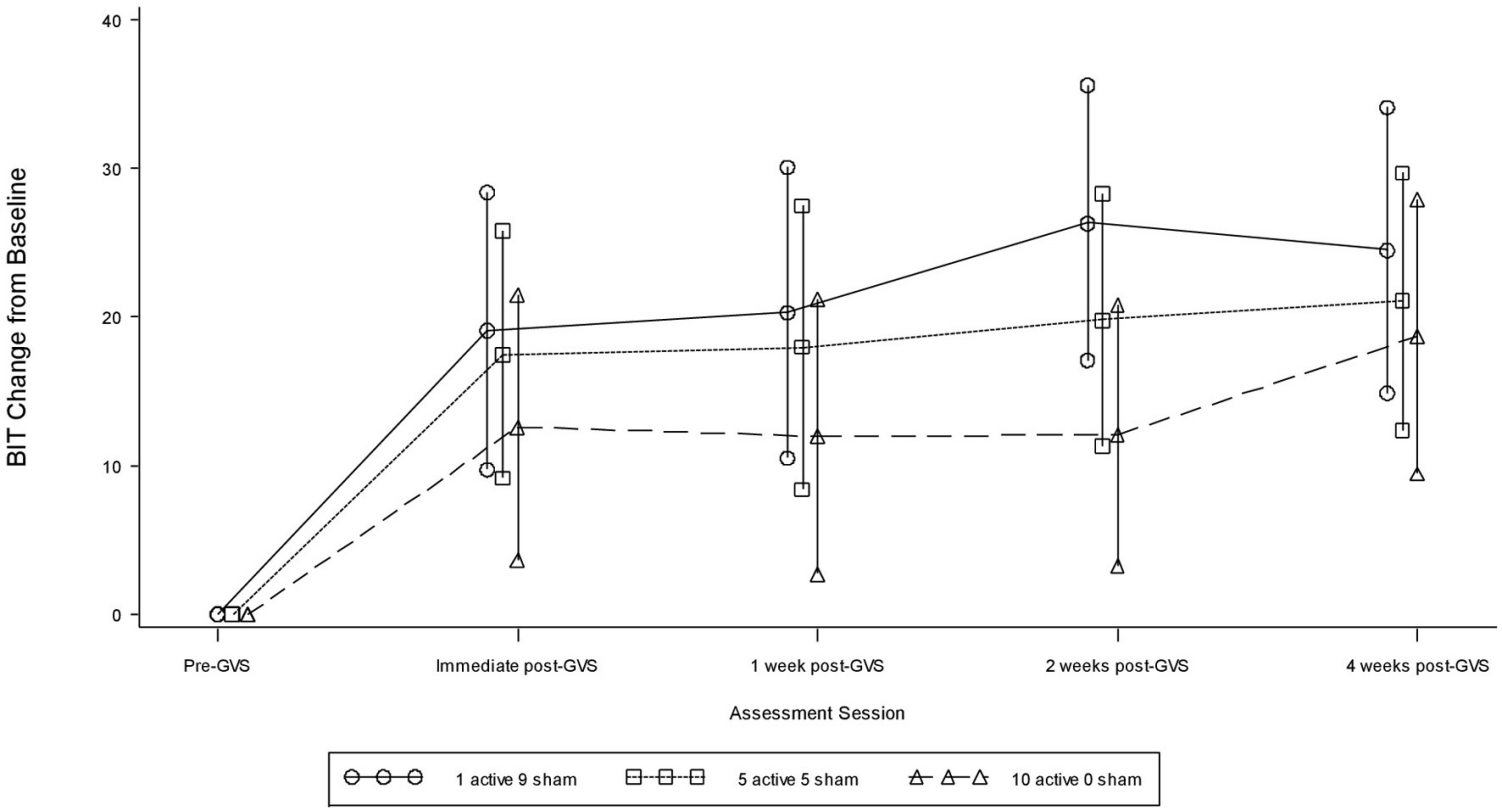
**Adjusted BIT mean scores and 95% confidence intervals showing change from baseline**.

Table 2 presents measures of central tendency and dispersion for each treatment arm. Table 3 shows the *p*-values from the corresponding ANCOVA of the adjusted mean scores, and highlights a significant association between mean BIT score at baseline and all subsequent sessions. Adjusted mean BIT scores (change from baseline) and corresponding 95% confidence intervals are shown in Figure 2, and indicate that the change in BIT score between baseline and 4 weeks post-GVS was statistically significant in all treatment arms. These changes were associated with large effect sizes: Cohen's *d* for 1 active, 5 active, and 10 active arms = 0.97, 1.29, and 1.48, respectively. The pattern of non-overlapping/overlapping confidence intervals in Figure 2 also indicate that the BIT scores at all other time-points were statistically different from baseline, although there were no statistically significant differences between treatment arms (see Table 4). The improvement in overall BIT performance from baseline to week 4 was evident on all sub-tests in all treatment arms, except for figure/shape copying in the 1 active arm, and free drawing in the 5 and 10 active arms (see Table 5). The differences across treatment arms in BIT sub-test scores at baseline and week 4 are depicted in Table 6, and although some appear to be marked, the statistical analysis did not show statistically significant differences. Likewise, the exploratory analysis indicated that Time since Stroke did not significantly affect outcome (see Tables 7, 8).

**Table 2:** **Summary of BIT scores**.

		**Treatment regimen**		
		**1 Active**	**5 Active**	**10 Active**
Baseline	Mean	73.3	78.7	85.0
	*SD*	40.2	34.0	35.9
	Median	81	83	97
	Minimum	13	15	28
	Maximum	124	125	124
	Range	111	110	96
	*N*	15	18	16
Session 10	Mean	92.9	96.3	97.4
	*SD*	38.9	36.0	38.1
	Median	97	110	113
	Minimum	21	37	17
	Maximum	143	142	140
	Range	122	105	123
	*N*	15	18	16
Week 1	Mean	94.9	104	96.9
	*SD*	37.3	31.7	38.7
	Median	108	122	106.5
	Minimum	15	53	21
	Maximum	143	143	139
	Range	128	90	118
	*N*	15	15	16
Week 2	Mean	101	102	97.1
	*SD*	36.7	34.1	36.0
	Median	111	119	106
	Minimum	19	53	31
	Maximum	145	146	138
	Range	126	93	107
	*N*	15	17	16
Week 4	Mean	99.6	99.9	104
	*SD*	39.8	35.8	39.3
	Median	111	113	122
	Minimum	19	28	26
	Maximum	146	144	142
	Range	127	116	116
	*N*	15	18	16
AUC (0–4 weeks)	Mean	393	424	395
	*SD*	150	127	147
	Median	430	489	460
	Minimum	73	229	102
	Maximum	578	577	545
	Range	505	348	443
	*N*	15	15	16

AUC, area under the curve.

**Table 3:** **Results from statistical analysis of BIT**.

	**Analysis of covariance (*p*-values)**				
	**Treatment**	**Treatment (linear)**	**Baseline**	**Age**	**Patient status**
Week 4	0.678	0.383	<0.001	0.0784	0.0670
AUC (0–4 weeks)	0.254	0.0964	<0.001	0.0943	0.360
Session 10	0.566	0.297	<0.001	0.0533	0.645
Week 1	0.432	0.200	<0.001	0.39	0.584
Week 2	0.0865	0.0261	<0.001	0.0203	0.330

**Table 4:** **BIT adjusted means and 95% confidence intervals—treatment differences**.

	**Adjusted means (95% confidence intervals)**		
	**Treatment difference**		
	**5 Active – 1 Active**	**10 Active – 1 Active**	**10 Active – 5 Active**
Week 4	−3.44 (−16.4 to 9.49)	−5.83 (−19.1 to 7.48)	−2.38 (−15.0 to 10.2)
AUC (0−4 weeks)	−15.0 (−64 to 33.9)	−39.1 (−86.7 to 8.53)	−24.1 (−71.2 to 23.1)
Session 10	−1.57 (−14.1 to 10.9)	−6.48 (−19.3 to 6.38)	−4.92 (−17.1 to 7.28)
Week 1	−2.35 (−16.2 to 11.5)	−8.35 (−21.8 to 5.09)	−5.99 (−19.3 to 7.30)
Week 2	−6.54 (−19.1 to 6.06)	−14.3 (−27.0 to −1.59)	−7.76 (−20.0 to 4.44)

**Table 5:** **Median scores on BIT sub-tests as function of number of active sessions**.

**No. active**	**BIT sub-tests**																	
	**Star cancellation (max = 54)**			**Letter cancellation (max = 40)**			**Line bisection (max = 9)**			**Line crossing (max = 36)**			**Copying (max = 4)**			**Free drawing (max = 4)**		
	1	5	10	1	5	10	1	5	10	1	5	10	1	5	10	1	5	10
Baseline	24	29	35	19	24	26	3	3	2	29	24	35	1	1	1	0	2	2
Session10	31	39	42	25	31	32	4	3	4	35	35	36	2	1	2	2	2	2
Week 1	42	36	39	24	30	26	4	4	4	33	35	35	1	2	2	2	2	3
Week 2	45	45	38	28	28	30	4	4	3	36	36	36	1	2	2	2	2	2
Week 4	47	38	42	25	33	33	4	6	5	35	34	36	1	2	2	2	2	2
Week 4-baseline	23	9	7	6	9	7	1	3	3	6	10	1	0	1	1	2	0	0

Parenthesized values denote maximum possible score. The bottom row shows change from baseline to week 4.

**Table 6:** **Inter-arm differences in median scores on BIT sub-tests at baseline and week 4**.

**No. active**	**BIT sub-tests**																	
	**Star cancellation**			**Letter cancellation**			**Line bisection**			**Line crossing**			**Copying**			**Free drawing**		
	**1–5**	**1–10**	**5–10**	**1–5**	**1–10**	**5–10**	**1–5**	**1–10**	**5–10**	**1–5**	**1–10**	**5–10**	**1–5**	**1–10**	**5–10**	**1–5**	**1–10**	**5–10**
Baseline	−5	−11	−6	−5	−7	−2	0	1	1	5	−6	−11	0	0	0	−2	−2	0
Week 4	9	5	−4	−8	−8	0	−2	−1	1	1	−1	−2	−1	−1	0	0	0	0

To illustrate, for star cancellation the median score at baseline was 5 points lower in the 1 active arm compared to the 5 active arm. By week 4, the median score was 9 points higher in the 1 active arm compared to the 5 active arm.

**Table 7:** **Results from the exploratory statistical analysis of BIT including time since stroke as a co-variate**.

	**Analysis of covariance (*p*-values)**					
	**Treatment**	**Treatment (linear)**	**Baseline**	**Age**	**Patient status**	**Time since stroke**
Week 4	0.733	0.542	<0.001	0.027	0.210	0.714
AUC (0–4 weeks)	0.352	0.196	<0.001	0.061	0.369	0.064

**Table 8:** **BIT adjusted means and 95% confidence intervals from the exploratory analysis including time since stroke as a co-variate—treatment differences**.

	**Adjusted means (95% Confidence intervals) Treatment difference**		
	**5 Active – 1 Active**	**10 Active – 1 Active**	**10 Active – 5 Active**
Week 4	−4.95 (−18.7 to 8.85)	−4.46 (−18.6 to 9.69)	0.488 (−13.4 to 14.3)
AUC (0−4 weeks)	−29.7 (−82.1 to 22.6)	−33.3 (−83.2 to 16.5)	−3.59 (−54.9 to 47.1)

### Barthel index

**Figure 3:**
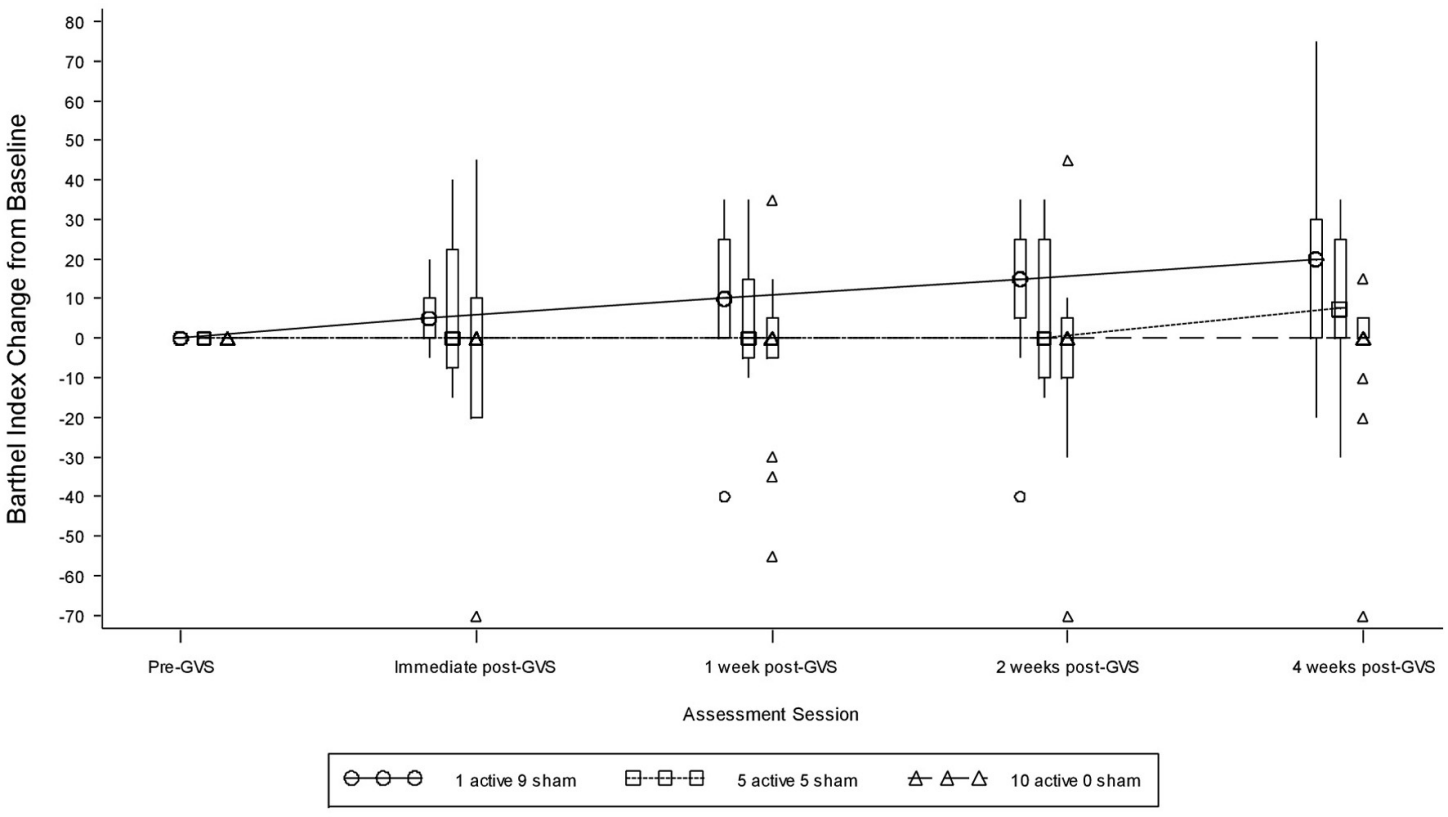
**Adjusted Barthel Index median scores showing change from baseline**. The boxes represent the inter-quartile range and are intersected at their median point. The whiskers extend to the most extreme point within 1.5 times of the inter-quartile range.

**Figure 4:**
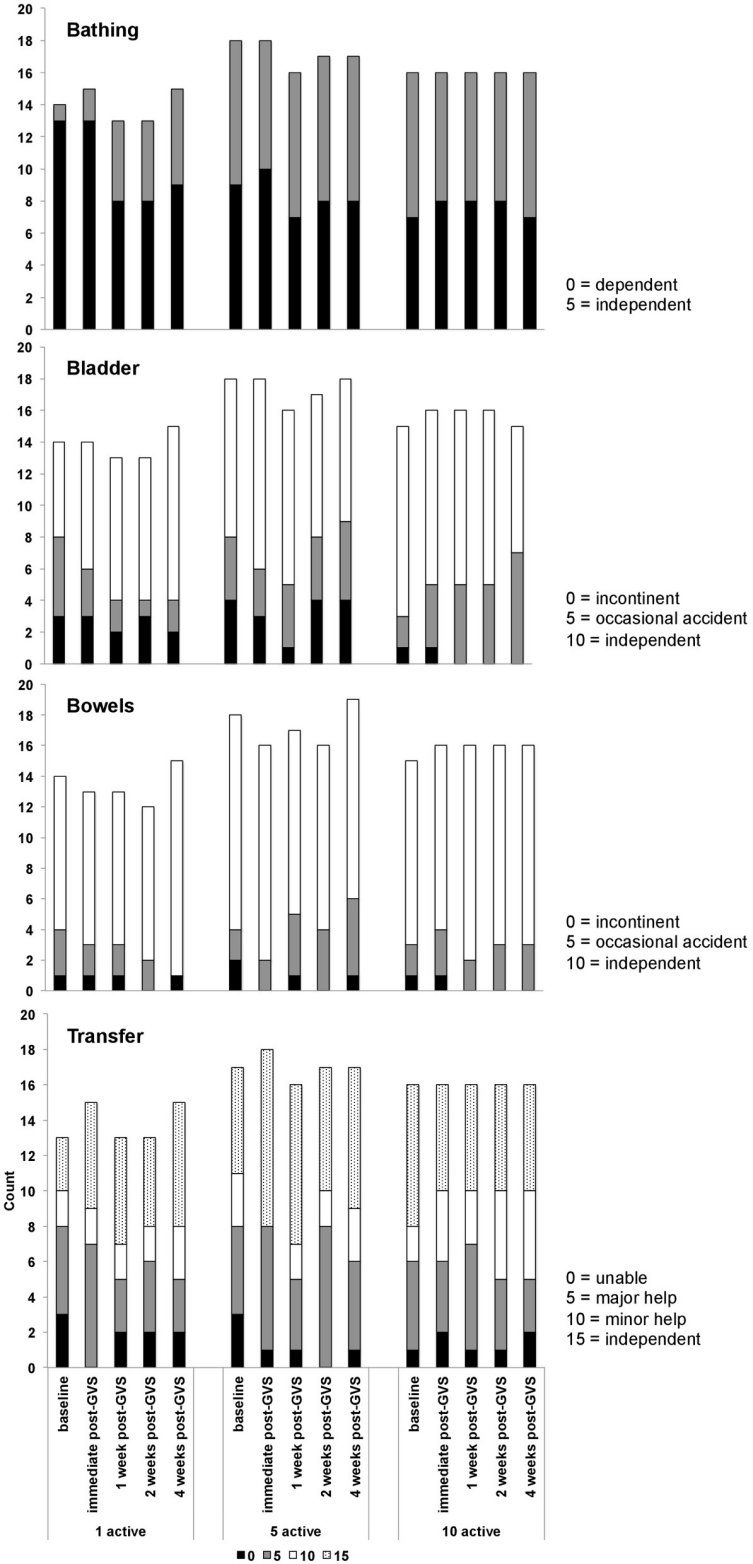
**Barthel index data showing the numbers of participants within each treatment arm who scored 0, 5, 10, or 15 on the bowels, bladder, bathing, and transfer sub-scales**.

Table 9 presents measures of central tendency and dispersion for each treatment arm. Table 10 shows the *p*-values for the corresponding ANCOVA and highlights a significant association between baseline BI score and the subsequent sessions. All adjusted BI median scores (change from baseline) and corresponding 95% confidence intervals are shown in Figure 3, and indicate that the change in BI median score between baseline and 4 weeks post-GVS was statistically significant in the 1 active treatment arm (though analysis of the ranked data showed that this change was also statistically significant in both other treatment arms). The pattern of overlapping confidence intervals indicates that there were no reliable differences between treatment arms. Summary data for the 1 active condition indicated that improvement was most evident on the bathing, bladder, bowels, and transfer (from bed to chair) sub-scales (see Figure 4). As with the BIT data, the exploratory analysis indicated that Time since Stroke did not significantly affect outcome (see Table 11).

**Table 9:** **Summary of BI scores**.

		**Treatment regimen**		
		**1 Active**	**5 Active**	**10 Active**
Baseline	Mean	42.9	53.8	71.3
	*SD*	27.4	32.8	26.8
	Median	40	50	80
	Minimum	0	5	20
	Maximum	90	100	100
	Range	90	95	80
	***N***	12	16	15
Session 10	Mean	49.6	60.8	62.3
	*SD*	24.1	29.1	31.0
	Median	50	70	65
	Minimum	5	20	15
	Maximum	75	100	100
	Range	70	80	85
	***N***	13	18	15
Week 1	Mean	59.1	65.3	61.1
	*SD*	27.3	27.4	21.9
	Median	70	70	55
	Minimum	0	20	25
	Maximum	85	100	100
	Range	85	80	75
	***N***	11	15	14
Week 2	Mean	57.5	53.2	63.3
	*SD*	30.9	27.4	25.9
	Median	60	45	65
	Minimum	5	15	25
	Maximum	95	95	100
	Range	90	80	75
	***N***	12	14	15
Week 4	Mean	64.3	56.9	66.4
	*SD*	24.5	25.6	26.5
	Median	67.5	57.5	70
	Minimum	0	15	25
	Maximum	90	100	100
	Range	90	85	75
	***N***	14	16	14

**Table 10:** **Results from statistical analysis of Barthel Index**.

	**Analysis of covariance (*p*-values)**				
	**Treatment**	**Treatment (linear)**	**Baseline**	**Age**	**Patient status**
Week 4	0.361	0.172	<0.001	0.568	0.709
Session 10	0.494	0.581	<0.001	0.338	0.746
Week 1	0.566	0.405	<0.001	0.591	0.288
Week 2	0.894	0.650	<0.001	0.426	0.687

**Table 11:** **Results from exploratory statistical analysis of Barthel Index including time since stroke as a co-variate**.

	**Analysis of covariance (*p*-values)**					
	**Treatment**	**Treatment (linear)**	**Baseline**	**Age**	**Patient status**	**Time since stroke**
Week 4	0.406	0.178	<0.001	0.630	0.679	0.934

### Adverse events and participant satisfaction

Table 12 presents summary data collected from participants' diary cards before and during the stimulation period. Relative to baseline, there was little evidence in any treatment group of increased sickness, headache, tiredness, dizziness, pain behind ears or visual disturbance. Participants in all treatment arms reported favorable opinions on the satisfaction questionnaire (see Table 13).

**Table 12:** **Summary of participant diary data**.

	**1 Active**			**5 Active**			**10 Active**		
	**Baseline**	**Days 1–5**	**Days 6–10**	**Baseline**	**Days 1–5**	**Days 6–10**	**Baseline**	**Days 1–5**	**Days 6–10**
**SICKNESS**									
Not at all	7	9	9	10	10	9	10	8	8
A little	2	0	0	1	0	1	0	1	1
Moderately	0	0	0	0	0	0	0	0	0
Very much	0	0	0	0	0	0	0	1	0
**HEADACHE**									
Not at all	6	7	9	5	7	8	9	7	6
A little	1	1	0	4	1	0	0	0	2
Moderately	0	1	0	2	1	0	1	3	0
Very much	0	0	0	0	1	2	0	0	1
**TIREDNESS**									
Not at all	1	1	1	6	6	5	1	0	0
A little	0	1	3	0	1	1	1	3	5
Moderately	4	5	4	3	1	2	5	3	2
Very much	3	2	1	2	2	2	3	4	2
**DIZZINESS**									
Not at all	8	9	9	11	10	9	10	9	7
A little	0	0	0	0	0	0	0	1	1
Moderately	0	0	0	0	0	1	0	0	1
Very much	0	0	0	0	0	0	0	0	0
**PAIN BEHIND EARS**									
Not at all	8	9	9	11	10	10	10	9	8
A little	0	0	0	0	0	0	0	1	0
Moderately	0	0	0	0	0	0	0	0	1
Very much	0	0	0	0	0	0	0	0	0
**VISUAL DISTURBANCE**									
Not at all	13	12	12	12	13	12	10	9	11
A little	1	2	1	0	0	0	2	4	2
Moderately	0	0	0	1	1	1	1	0	0
Very much	0	0	0	1	0	1	1	1	1

**Table 13:** **Participant satisfaction questionnaire**.

**Question**	**Response**	**Treatment regimen**		
		**9 Sham 1 Active**	**5 Sham 5 Active**	**10 Active**
1. How has the course of treatment affected your awareness of your surroundings?	Much better	4	7	4
	A little better	6	7	7
	No different	3	3	3
	A little worse	0	0	0
	Much worse	0	0	0
2. In an overall, general sense how has the treatment made you feel about yourself?	Much better	3	5	2
	A little better	3	2	6
	No different	7	9	6
	A little worse	0	1	0
	Much worse	0	0	0
3. Was the treatment worth the effort of attending all the sessions?	Definitely yes	12	14	11
	Probably yes	1	3	2
	Not sure	0	0	1
	Probably no	0	0	0
	Definitely no		0	0
4. Are you happy with how the researchers behaved toward you?	Definitely yes	13	18	14
	Probably yes	0	0	0
	Not sure	0	0	0
	Probably no	0	0	0
	Definitely no	0	0	0
5. If a friend were in need of similar help, would you recommend our treatment to him or her?	Definitely yes	12	18	9
	Probably yes	1	0	4
	Not sure	0	0	1
	Probably no	0	0	0
	Definitely no	0	0	0

Values denote participant counts.

## Discussion

The marked improvement in mean BIT scores observed straight after the last stimulation session was still evident 4 weeks later. The scores recorded in this final follow-up were, for all treatment arms combined, 28% greater than those at baseline and gave rise to a large effect size (Cohen's *d* >1.0). This improvement was observed within all treatment arms, was evident on all BIT sub-tests and was not affected by time since stroke. For participants in the single treatment arm, improvement transferred beyond the diagnostic measure of the BIT to the BI, a widely used, albeit relatively crude, measure of activities of daily living. Here there was a median improvement of 20%—a change considered to be clinically important, with changes most noticeable on the continence, bathing and transfer sub-scales. These changes were achieved in the absence of any reported adverse events and at a high level of participant compliance and satisfaction.

The comparable efficacy of a single versus multiple stimulation sessions is perhaps surprising given that most forms of cognitive rehabilitation rely on repeated administration. However, as mentioned in the Introduction, three, small GVS studies have shown carry-over from a single session (Kerkhoff et al., 2011; Schmidt et al., 2013; Zubko et al., 2013). This may partly stem from the fact that the vestibular nerve is stimulated many thousands of times during a single 25–30 min session. This rate of stimulus repetition is much higher than that achieved with conventional behavioral interventions such as visual scanning therapy and contralesional limb activation and may, over a single session, be sufficient to induce long-term change in synaptic transmission (see Cooke and Bliss, 2006). The fact that these changes can be preferentially lateralized to the lesioned hemisphere via bipolar, binaural GVS may be particularly relevant given that neglect is associated with chronic under- and over-activation of the right and left hemisphere attentional systems respectively (Kinsbourne, 1977). The propensity for cortical change may be further enhanced by the distal up-regulation of key neurotransmitters within the brainstem during vestibular stimulation. Increased concentration of glutamate, a transmitter deemed especially important for NMDA-mediated synaptic excitability, has been observed within ascending pathways of the parabrachial nuclei and solitary tract during stimulation (Cai et al., 2007). Allied changes in serotonin release from the medial vestibular nuclei (Ma et al., 2007) and acetylcholine from hippocampal structures (Horii et al., 1994) may further facilitate recovery by heightening general arousal and alleviating co-morbid affective and cognitive disorders (Wilkinson et al., 2012).

On a cautionary note, the absence of a contemporaneous no-stimulation condition raises the question as to whether the improvement reported here was simply the result of natural recovery, practice and/or placebo. Such accounts cannot yet be ruled out with certainty. We chose not to include a no-stimulation condition because differences were expected between the treatment arms which would, given the blinding and minimization procedures employed, be sufficient to attribute at least some of the carry-over to GVS and thereby demonstrate proof-of-concept. Although treatment differences were not found, we believe it too coincidental for so many of the participants' natural recovery to be time-locked to the ~2 week period between baseline assessment and the final day of stimulation, not least given their sub-acute and chronic status (recall that all patients were at least 6 weeks post-onset). If the initial improvement reflected increased familiarity with the test materials then, contrary to the results, one might have expected further improvement at the later sessions. Against a general practice effect, we also note that the test/retest reliability of the BIT across sessions spaced approximately 2 weeks apart (i.e., the time window in which most recovery occurred here) is high, yielding a correlation of 0.99 (Wilson et al., 1987). Regarding the potential influence of placebo, other neurostimulation studies have reported minimal placebo effects within this population (Nyffeler et al., 2009; Cazzoli et al., 2012; Koch et al., 2012). Aside from the use of blinding to counter placebo effects, the general absence of a strong placebo is also taken to reflect neglect patients' characteristic lack of affect and self-awareness. It also seems unlikely that any placebo occurring straight after stimulation would persist with the same intensity 1 month later, as observed here. Nevertheless, if this was the case then such a powerful placebo is, in its own right, worthy of further clinical investigation.

Aside from including a no-stimulation condition, we propose that further study should incorporate longer-term follow-up assessments. It remains possible that a greater number of sessions are more efficacious than a single one, but that longer follow-ups, perhaps in the order of months rather than weeks, are needed before this advantage becomes apparent. A second design issue concerns the best current amplitude to apply. We chose a 1 mA waveform because this is usually subliminal in older stroke patients yet known to modulate relevant neurophysiological and visual functions (Wilkinson et al., 2005, 2008, 2010, 2012; Zubko et al., 2013). But studies that perturb the vestibular stimulation via the more potent stimulus of ice-cold irrigation of the external ear canal have tended to eliminate (albeit transiently) rather than merely reduce neglect (Rubens, 1985; Cappa et al., 1987). The implication is that greater electrical currents may exert stronger relief than observed. The problem is that greater currents induce distracting side-effects, such as nausea and vertigo, and increase the risk of electrode burn. Future study therefore needs to establish if higher currents affect patient compliance within an acceptable margin. To this end, Utz et al. (2011b) recently demonstrated that, despite increased mild itching and tingling at the electrode sites, neglect patients were just as willing to receive GVS at 1.5 mA (super-sensory) as 0.6 mA. A key question is whether this willingness persists at even higher and potentially more efficacious levels. A final recommendation for future study is to incorporate multiple baseline assessments to better capture the rate of natural recovery. We excluded these assessments because the patients were sub-acute and the rate of natural recovery was assumed to be broadly comparable across treatment arm. But such repeat assessments must be included if studies are to now move beyond proof-of-concept and more accurately estimate treatment effect.

In closing, the current data endorse the growing sense that non-invasive neurostimulation may offer a viable alternative to pharmacological and behavioral interventions for neglect (Utz et al., 2010; Oliveri, 2011). Most neurostimulation research has focused on the potential benefits of transcranial direct current stimulation and transcranial magnetic stimulation. These techniques have also shown preliminary efficacy in neglect patients (see Müri et al., 2013). As in the present study, one recent TMS trial administered 10 daily sessions of stimulation and showed comparable improvement (23%) in BIT scores at 1 month follow-up (Koch et al., 2012). A subsequent study found that just 2 sessions of theta burst activity were sufficient to induce improvement for up to 3 weeks on the Catherine Bergego cale (Cazzoli et al., 2012), a measure of activities of daily living (Azouvi et al., 2003). However, the clinical application of these allied techniques still lacks systematic investigation—stimulation protocols need to be finessed, mechanistic bases elucidated, and too few studies incorporate adequate sample sizes, long follow-ups and measures of functional transfer (Müri et al., 2013; Yang et al., 2013). One advantage of GVS over these other stimulation methods is that delivery is simpler because there is no uncertainty about where on the scalp to apply stimulation—the electrodes are simply fastened to the mastoid processes. There is also no reported increase in seizure risk—if anything vestibular stimulation may reduce the likelihood of seizure onset (Kantner et al., 1982). In addition, GVS is cheap (relying on just a small battery, a simple micro-processor that can manage several stimulation parameters, two leads and a pair of electrodes), portable and suitable for home-based administration. Given the empirical data reported in the current study, we therefore recommend a further stage of optimization and efficacy testing before direct comparisons are made between GVS and these other emerging treatment options.

### Conflict of interest statement

The authors declare that the research was conducted in the absence of any commercial or financial relationships that could be construed as a potential conflict of interest.
